# Identification of potential crucial genes and key pathways in osteosarcoma

**DOI:** 10.1186/s41065-020-00142-0

**Published:** 2020-07-14

**Authors:** Junwei Liu, Siyu Wu, Xiaoyu Xie, Ziming Wang, Qianqian Lei

**Affiliations:** 1 Department of Orthopedic surgery, Daping Hospital, Army medical university, No. 10 Changjiang Branch Road, Yuzhong District, Chongqing, 400042 PR China; 2 Department of Radiation Oncology, Chongqing University Cancer Hospital, No. 181, Hanyu road, Shapingba District, Chongqing, 400030 PR China

**Keywords:** Osteosarcoma, Gene expression omnibus, Differentially expressed genes, Bioinformatics, Pathway

## Abstract

**Background:**

The aim of this study is to identify the potential pathogenic and metastasis-related differentially expressed genes (DEGs) in osteosarcoma through bioinformatic analysis of Gene Expression Omnibus (GEO) database.

**Results:**

Gene expression profiles of GSE14359, GSE16088, and GSE33383, in total 112 osteosarcoma tissue samples and 7 osteoblasts, were analyzed. Seventy-four normal-primary DEGs (NPDEGs) and 764 primary-metastatic DEGs (PMDEGs) were screened. VAMP8, A2M, HLA-DRA, SPARCL1, HLA-DQA1, APOC1 and AQP1 were identified continuously upregulating during the oncogenesis and metastasis of osteosarcoma. The enriched functions and pathways of NPDEGs include procession and presentation of antigens, activation of MHC class II receptors and phagocytosis. The enriched functions and pathways of PMDEGs include mitotic nuclear division, cell adhesion molecules (CAMs) and focal adhesion. With protein-protein interaction (PPI) network analyzed by Molecular Complex Detection (MCODE) plug-in of Cytoscape software, one hub NPDEG (HLA-DRA) and 7 hub PMDEGs (CDK1, CDK20, CCNB1, MTIF2, MRPS7, VEGFA and EGF) were eventually selected, and the most significant pathways in NPDEGs module and PMDEGs module were enriched in the procession and presentation of exogenous peptide antigen via MHC class II and the nuclear division, respectively.

**Conclusions:**

By integrated bioinformatic analysis, numerous DEGs related to osteosarcoma were screened, and the hub DEGs identified in this study are possibly part of the potential biomarkers for osteosarcoma. However, further experimental studies are still necessary to elucidate the biological function and mechanism of these genes.

## Introduction

Osteosarcoma is the most prevalent primary bone malignancy and the 8th most frequent type of malignancy that disproportionally affects children and young adults [1]. In recent decades, the improvement in osteosarcoma’s treatment (surgery and chemotherapy) has largely increased the long-term survival rate (approximately 60–70%) of children and young adult patients with osteosarcoma without distal metastasis [2, 3]. However, the etiology remains unknown, and this discourages the prevention and early diagnosis of osteosarcoma. Therefore, it is extremely necessary to explore the mechanisms behind the occurrence and progression of osteosarcoma.

In recent years, the development in molecular biology has provided some new insights into the potential diagnostic and therapeutic biomarkers for osteosarcoma [4]. Genome-wide molecular profiling, which reveals molecular changes in tumorigenesis and progression, has been proved to be an efficient approach to identify key genes [5, 6]. However, it requires considerable time and fund to obtain clinical biological samples and subsequently conduct high-throughput genetic detection and analysis. Cumulative studies in the past have shown that re-analyzing gene datasets of previous experiments from online databases is a feasible way to find out biologically and clinically relevant biomarkers (genes) [7–11], and that some of those biomarkers (genes) have even been found to play important roles in osteosarcoma. For instance, by conducting bioinformatics analysis on three datasets deposited in GEO database (GSE36001, GSE19276 and GSE16088), Pan Liu et al. [10] revealed that tumor protein p53 (TP53), mitogen-activated protein kinase 1 (MAPK1), estrogen receptor 1 (ESR1), notch homolog protein 3 (NOTCH3) and caspase 1 (CASP1) might potentially be important osteosarcoma-associated genes. Among them, mutant TP53 was subsequently reported to be associated with poor survival of osteosarcoma patients, because it can increase the cell proliferation, migration, and chemoresistance in osteosarcoma [12]; MAPK1 has been confirmed to be highly expressed in osteosarcoma cells, and can be down-regulated by osteosarcoma related tumor suppressive miR-511 [13]. Based on this, regulation of MAPK1 receptor expression may be a novel approach to treat osteosarcoma. Not long ago, Notch3 overexpression also was confirmed to be associated with metastasis and poor prognosis in osteosarcoma patients [14]. All those examples suggest that bioinformatics analysis is a feasible approach to identify specific genes that may provide valuable clues for investigating the pathogenesis of osteosarcoma.

The current study aims to investigate the crucial genes and key pathways potentially involved in osteosarcoma tumorigenesis and development. To achieve this, we integrated bioinformatics analysis based on Gene Expression Omnibus (GEO) datasets. The data obtained indicate that some genes might continue to participate in the occurrence and metastasis of osteosarcoma.

## Materials and methods

### Osteosarcoma datasets

The following criteria were applied to screen out appropriate gene expression data: i) primary or metastatic osteosarcoma tissues were included as tumor samples; ii) normal human bone samples or human osteoblasts were included as normal counterparts; iii) more than 1000 DEGs with FDR (i.e. adjusted *p*-value) < 0.05 and |log2fold-change (FC)| > 1 as the cut-off criteria [7–11]; and iv) more than 10 overlapping DEGs with other datasets. Three datasets were finally included. Dataset GSE14395 [15] contains 5 frozen osteosarcomas (from 5 patients, 27.2 ± 24.0 years, 2 females and 3 males) and 4 osteosarcoma lung metastasis samples (from 4 patients, 35.8 ± 9.0 years, 3 females and 1 males) and 1 fresh primary osteoblast cell HOBc (two duplicates for each sample). Dataset GSE16088 [16] contains 15 frozen osteosarcomas (clinical data was not available) and 3 fresh primary osteoblast cells (U2, HOS and MG63). Dataset GSE33383 [17] contains 82 osteosarcomas (from 84 patients, 19.0 ± 11.7 years, 29 females, 54 males and 1 gender unknown) and 3 fresh primary osteoblast cells (220-OB, 240-OB and Kaat-OB).

The three gene expression profiles above were downloaded from the Gene Expression Omnibus (GEO) database (https://www.ncbi.nlm.nih.gov/geo/) [18] for identification of DEGs. Detailed information of all datasets included is listed in Table 1.

**Table 1:** Characteristics of datasets in this study

Dataset	Platform	Sample			Country
		Normal	Primary tumor	Metastatic tumor	
GSE14359 [15]	Affymetrix HG U133A	1 osteoblasts (two duplication)	10 tissue samples	4 lung samples	Germany
GSE16088 [16]	Affymetrix HG U133A	3 osteoblasts (U2, HOS and MG63)	14 tissue samples		USA
GSE33383 [17]	Illumina human-6 v2.0	3 osteoblasts (220-OB, 240-OB and Kaat-OB)	84 tissue samples		Norway

### Data preprocessing

The analysis of raw probe-level data (.CEL files) was performed using the robust multiarray average algorithm (RMA) in the Affy package of R [19]. After background correction and quantile normalization, the expression values were obtained. The averages of the probe set of values were calculated as the expression values for the same gene with multiple probe sets [20].

### Identification of DEGs

Identification of DEGs was performed using the LIMMA package of R [21]. The adjusted *P*-values (adj P-value) were adopted to avoid the occurrence of false-positive results. Using the Benjamini-Hochberg method [22] via the multtest package in R, the FDR (that is, adjusted *p*-value) < 0.05 and |log2fold-change (FC)| > 1 were used as the cut-off criteria, as previously reported [7–11]. Online tool EHBIO ImageGP (http://www.ehbio.com/ImageGP) operated by EHBIO Gene Technology (Beijing) Co., Ltd. (Beijing, China) was applied to generate volcano plot and Venn diagram, respectively, for the visualization of the identified DEGs.

### Functional enrichment analysis

GO (Gene Ontology) function and KEGG (Kyoto Encyclopedia of Genes and Genomes) pathway enrichment analyses of the DEGs were performed using the clusterProfiler package of R [8]. The GO and KEGG terms with FDR < 0.05 were regarded as significant functions and pathways.

### Protein-protein interaction network construction and module analysis

The Search Tool for the Retrieval of Interacting Genes (STRING; http://string.embl.de/) is a database of protein-protein interactions known and predicted (PPIs) [23]. Based on the STRING online tool, PPIs of the DEGs were constructed with a confidence score ≥ 0.7. Subsequently, the PPI network was visualized by means of Cytoscape software (version 3.7.2). Furthermore, Molecular Complex Detection (MCODE) [24] plug-in in Cytoscape software was applied to explore the significant modules in PPI network. The advanced options set as degree cutoff = 2, K-Core = 2, and Node Score Cutoff = 0.2. Given that it’s hard to conduct enrichment analysis based on small gene sets with the clusterProfiler package of R, instead conducted was the GO function enrichment analysis of DEGs in each module using ClueGo [25] and CluePedia [26] plug-ins of Cytoscape software. The GO terms with FDR < 0.05 (Benjamini-Hochberg method) were regarded as significant functions.

## Results

### Identification of DEGs between normal osteoblasts and osteosarcoma samples

**Fig. 1:**
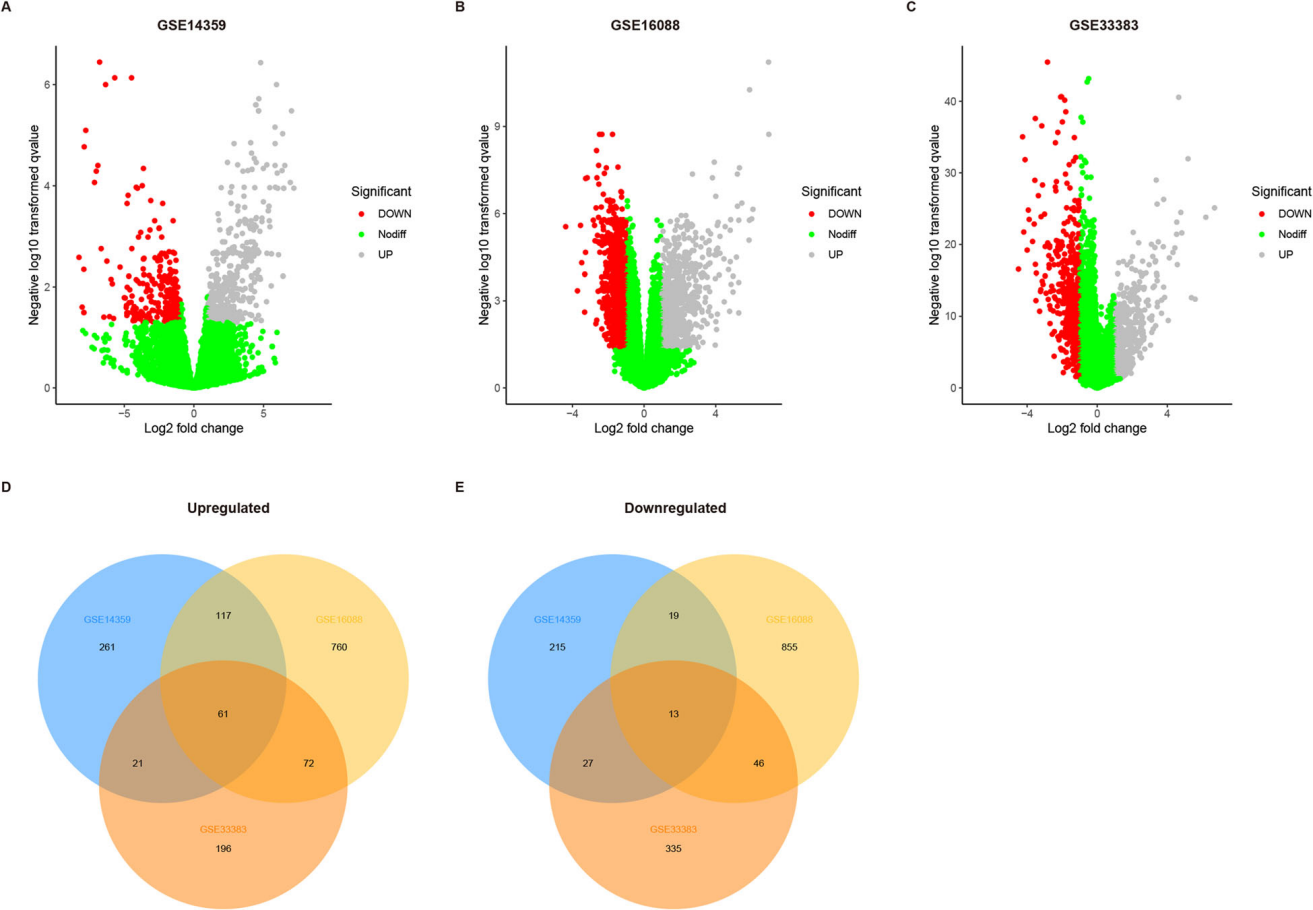
Identification of differentially expressed genes (DEG) between osteosarcoma cell lines and osteoblasts. **a-c** Volcano plots for expression of DEGs in dataset GSE14359 (**a**), GES16088 (**b**) and GSE33383 (**c**). Red dots represent upregulated DEGs, grey dots represent downregulated DEGs, and green dots represent DEGs with no differences. **d-e** The Venn diagrams of the overlapping DEGs among the three datasets

According to the screening criteria, this study enrolled three datasets (Table 1) to identify genes differentially expressed between normal osteoblasts and osteosarcoma tissue samples. There were 777 normal-primary DEGs (NPDEGs; 489 up-regulated and 288 down-regulated) in GSE14359, 1943 NPDEGs (1010 up-regulated and 933 down-regulated) in GSE16088, and 771 NPDEGs (350 up-regulated and 421 down-regulated) in GSE33383 (Fig. 1a–c). Further analysis of these NPDEGs with Venn diagram revealed that there were 61 up-regulated overlapping NPDEGs, and 13 down-regulated overlapping NPDEGs among all three datasets (Fig. 1d-e, Supplementary Table 1).

### Functional enrichment analysis of DEGs between Normal tissue and osteosarcoma samples

**Fig. 2:**
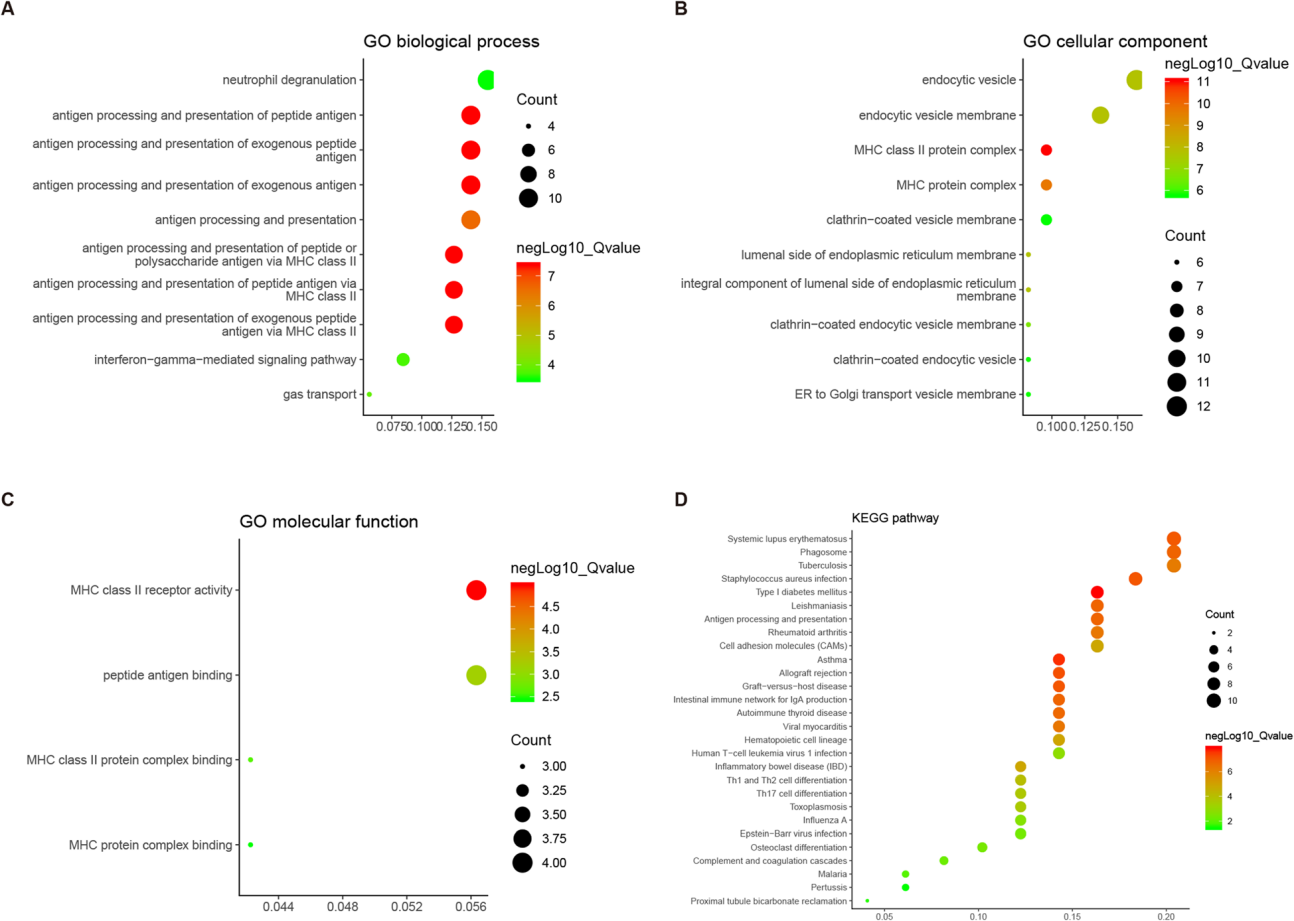
GO classification and KEGG pathway analysis of 74 normal-primary related DEGs. **a-c** GO enrichment analysis of NPDEGs in the biological process (**a**), cellular component (**b**), and molecular function (**c**) categories. **d** KEGG pathway analysis of NPDEGs. The X-axis represents the enrichment levels. The larger value of Rich factor represents the higher level of enrichment. The color of the dot stands for the different *P*-value and the size of the dot reflects the number of target genes enriched in the corresponding pathway. GO, Gene Ontology; KEGG, Kyoto Encyclopedia of Genes and Genomes

To further investigate the biological functions of the 74 NPDEGs, GO and pathway analysis were performed using the clusterProfiler package of R. GO analysis (Supplementary Table S2) showed that the NPDEGs between osteosarcoma and normal tissue samples were clustered in 82 significant biological process (BP) categories. As shown in Fig. 2a (top ten BP categories), most were clustered in antigen procession and presentation. NPDEGs were clustered in 43 significant cellular component (CC) categories. As shown in Fig. 2b (top ten CC categories), the most significant CC category was MHC class II protein complex. DEGs were clustered in 4 significant molecular function (MF) categories. As shown in Fig. 2c, the most significant MF category was MHC class II receptor activity. KEGG analysis identified 28 significant pathways, such as phagosome and antigen procession and presentation (Fig. 2b, Supplementary Table S2).

### Identification of DEGs between primary and metastatic osteosarcoma samples

**Fig. 3:**
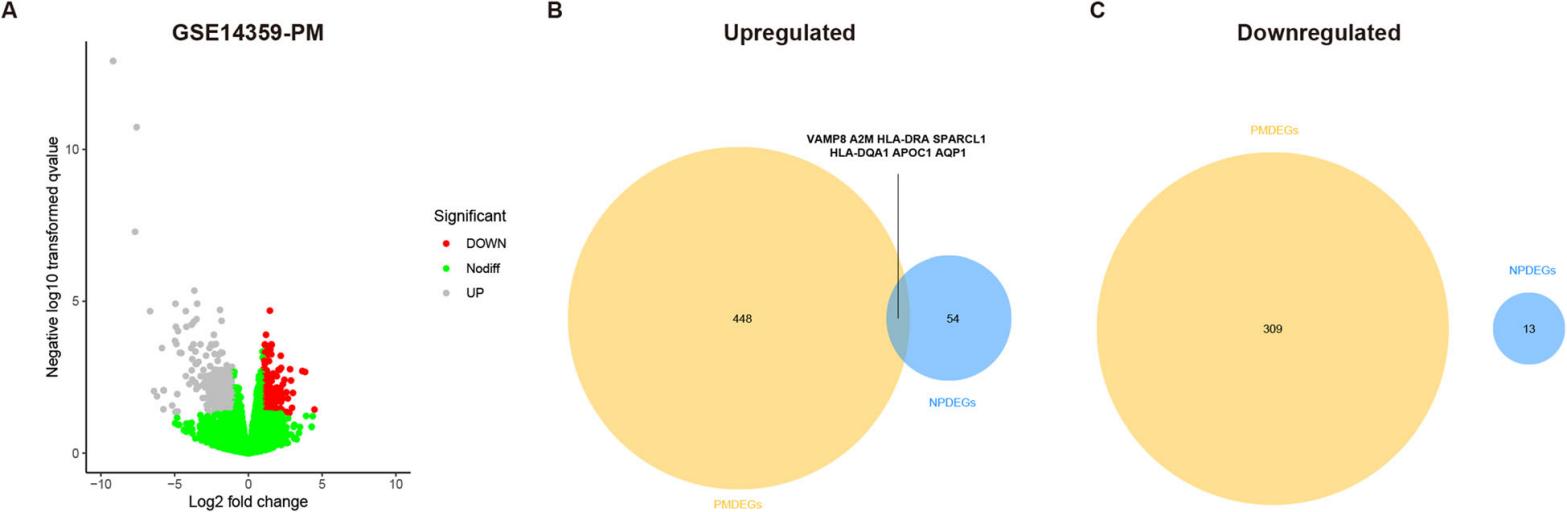
Identification of differentially expressed genes (DEG) between primary and metastatic osteosarcoma tissue samples. **a** Volcano plots for expression of PMDEGs in GSE14359 dataset. Red dots represent upregulated DEGs, grey dots represent downregulated DEGs, and green dots represent genes with no differences. **b** The Venn diagrams of the overlapping DEGs between the upregulating NPEGSs and PMDEGs. **c** The Venn diagrams of the overlapping DEGs between the downregulating NPEGSs and PMDEGs

Based on the screening criteria, only GSE14359 dataset was selected for identifying genes differentially expressed between primary and metastatic osteosarcoma samples. There were 764 primary-metastasis DEGs (PMDEGs, 309 up-regulated and 455 down-regulated) in GSE14359 (Fig. 3a and Supplementary Table S3). Interestingly, seven overlapping up-regulated DEGs (VAMP8, A2M, HLA-DRA, SPARCL1, HLA-DQA1, APOC1 and AQP1) were identified between the NPDEGs and PMDEGs (Fig. 3b, Table 2), whereas there was none overlapping down-regulated DEG (Fig. 3c). This suggests that these seven genes may act as oncogenes and continue to participate in the development and metastasis of osteosarcoma.

**Table 2:** DEGs continuous upregulating during the oncogenesis and metastasis of osteosarcoma

Gene symbol	Full name	GO BP ID	KEGG pathway
HLA-DRA	Major histocompatibility complex, class II, DR alpha	GO:0002478, GO:0019884, GO:0019886, GO:0048002, GO:0002495, GO:0002504, GO:0019882, GO:0060333, GO:0002429, GO:0071346, GO:0002768, GO:0034341, GO:0050852, GO:0002503, GO:0050851, GO:0002399, GO:0002501, GO:0002396	hsa04940, hsa05310, hsa05330, hsa05332, hsa05150, hsa05322, hsa04672, hsa05140, hsa04612, hsa04145, hsa05320, hsa05416, hsa05323, hsa05152, hsa04640, hsa05321, hsa04514, hsa04658, hsa04659, hsa05145, hsa05166, hsa05164, hsa05169
VAMP8	Vesicle Associated Membrane Protein 8	GO:0002478, GO:0019884, GO:0048002, GO:0019882, GO:0043312, GO:0002283, GO:0042119, GO:0002446, GO:0002697, GO:0002696, GO:0050867, GO:0002699, GO:0043304, GO:0033006, GO:0042590	/
A2M	Alpha-2-Macroglobulin	GO:0002697	hsa04610
SPARCL1	SPARC Like 1	/	/
HLA-DQA1	Major Histocompatibility Complex, Class II, DQ Alpha 1	GO:0002478, GO:0019884, GO:0019886, GO:0048002, GO:0002495, GO:0002504, GO:0019882, GO:0060333, GO:0002429, GO:0071346, GO:0002768, GO:0034341, GO:0050852, GO:0050851	hsa04940, hsa05310, hsa05330, hsa05332, hsa05150, hsa05322, hsa04672, hsa05140, hsa04612, hsa04145, hsa05320, hsa05416, hsa05323, hsa05152, hsa04640, hsa05321, hsa04514, hsa04658, hsa04659, hsa05145, hsa05166, hsa05164, hsa05169
APOC1	Apolipoprotein C1	GO:0043062, GO:0051346, GO:0060627, GO:0030100	/
AQP1	Aquaporin 1	GO:0015669, GO:0015701, GO:0046677, GO:0030185, GO:0006979, GO:0015670, GO:0048545, GO:0042476, GO:0042542, GO:0097237	hsa04964

**/:** no significant related GO BP or KEGG pathway term

### Functional enrichment analysis of DEGs between primary and metastatic osteosarcoma samples

**Fig. 4:**
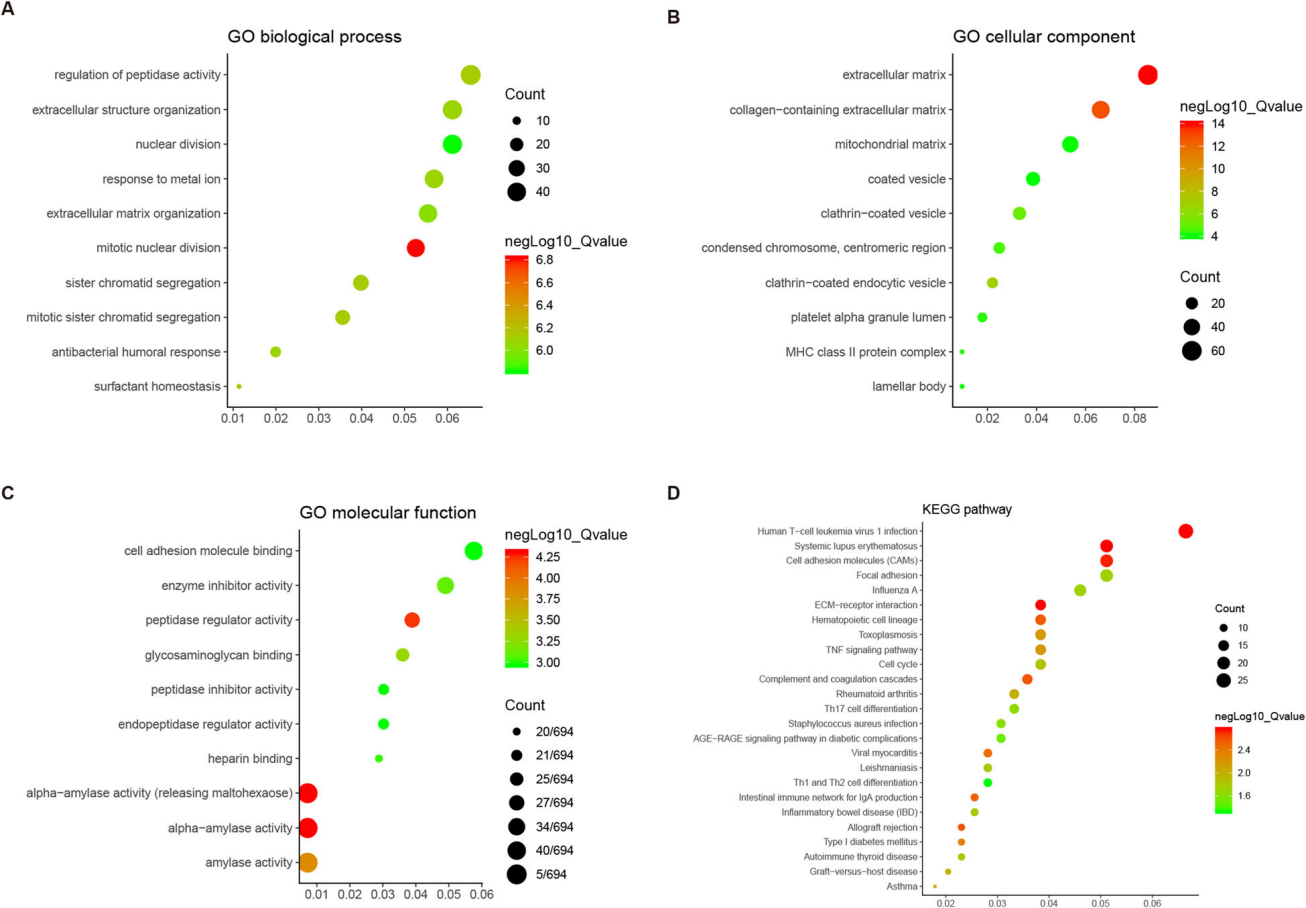
GO classification and KEGG pathway analysis of 764 PMDEGs. **a-c** GO enrichment analysis of PMDEGs in the biological process (**a**), cellular component (**b**), and molecular function (**c**) categories. **d** KEGG pathway analysis of PMDEGs. The X-axis represents the enrichment levels. The larger value of Rich factor represents the higher level of enrichment. The color of the dot stands for the different P-value and the size of the dot reflects the number of target genes enriched in the corresponding pathway. GO, Gene Ontology; KEGG, Kyoto Encyclopedia of Genes and Genomes

GO analysis (Supplementary Table S4) showed the 764 PMDEGs were clustered in 162 significant BP categories. As shown in Fig. 4a (top ten BP categories), the most significant BP category was mitotic nuclear division. PMDEGs were clustered in 57 significant cellular CC categories. As shown in Fig. 4b (top ten CC categories), the most significant CC category was extracellular matrix (ECM). PMDEGs were clustered in 16 significant molecular function (MF) categories. As shown in Fig. 4c (top ten MF categories), the most significant MF category was alpha-amylase activity. KEGG analysis identified 25 significant biological pathways, such as cell adhesion molecules (CAMs) and focal adhesion (Fig. 4d, Supplementary Table S4).

### PPI (protein-protein interaction) network and module analysis

**Fig. 5:**
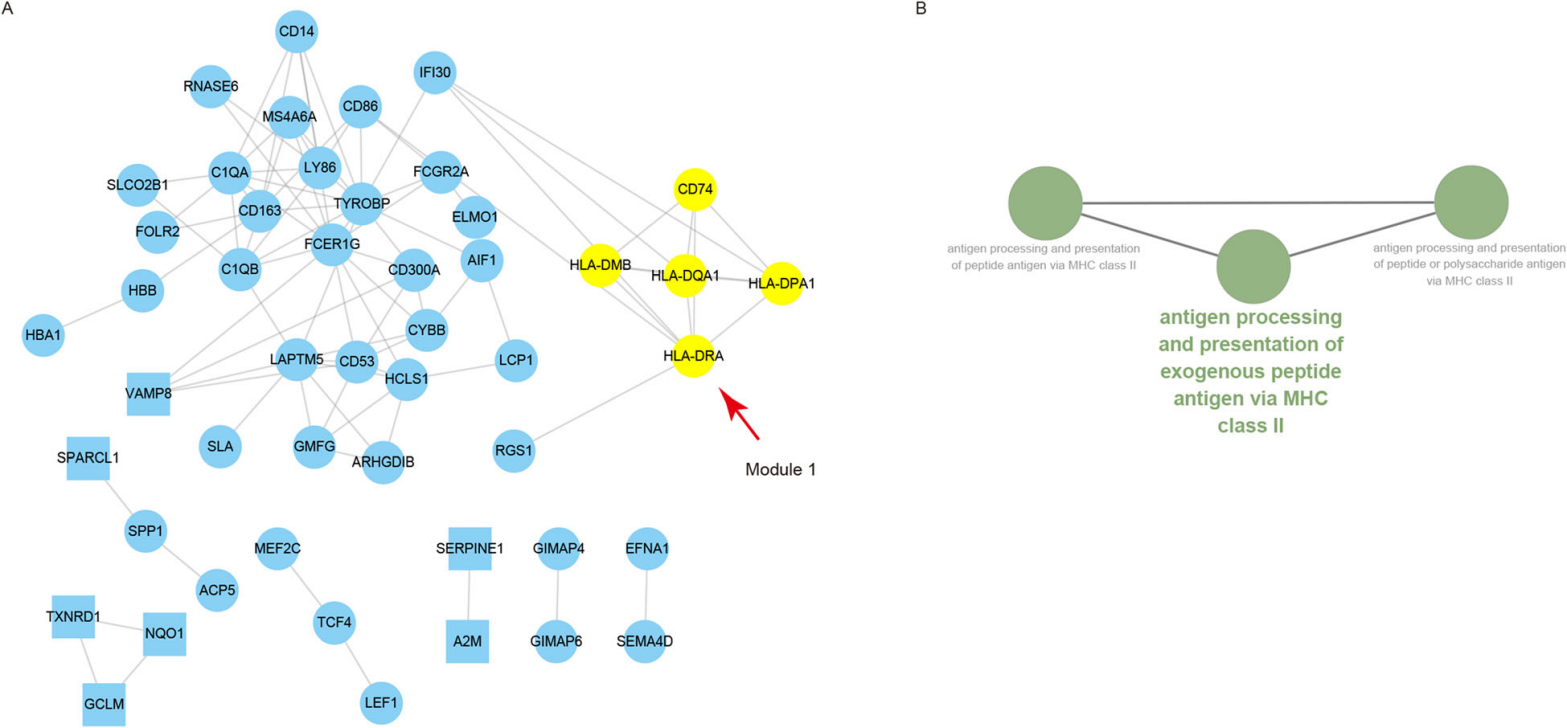
Protein-protein interaction (PPI) network construction and GO enrichment analysis of the 5 gene module. **a** PPI network constructed with the 74 NPDEGs. All cycle nodes stand for upregulated genes, and all square nodes stand for downregulated genes. Genes in module 1 are in yellow. The seed node lives at the center of module 1. **b** The biological process categories associated with genes in module 1 through GO enrichment analysis (FDR < 0.05). GO, Gene Ontology

PPI Network was subsequently analyzed and proteins were selected based on a combined score ≥ 0.7 in STRING analysis. There were 49 nodes and 91 interactions among the 74 NPDEGs (Fig. 5a, Supplementary Table S5). In addition, one significant module with a score = 5 was screened out via MCODE, and HLA-DRA was the hub gene with the highest degree of connectivity (Table 3). GO analysis with ClueGO showed that the most significant BP category in this module was enriched in the antigen processing and presentation of exogenous peptide antigen via MHC class II (Fig. 5b, Supplementary Table S6).

**Table 3:** Hub genes with high degree of connectivity in each module

Category	MCODE module	Gene symbol	Degree	Type	Full name	Go BP ID	KEGG pathways
NPDEG	Module 1	HLA-DRA	7	Up	Major histocompatibility complex, class II, DR alpha	GO:0002478, GO:0019884, GO:0019886, GO:0048002, GO:0002495, GO:0002504, GO:0019882, GO:0060333, GO:0002429, GO:0071346, GO:0002768, GO:0034341, GO:0050852, GO:0002503, GO:0050851, GO:0002399, GO:0002501, GO:0002396,	hsa04940, hsa05310, hsa05330, hsa05332, hsa05150, hsa05322, hsa04672, hsa05140, hsa04612, hsa04145, hsa05320, hsa05416, hsa05323, hsa05152, hsa04640, hsa05321, hsa04514, hsa04658, hsa04659, hsa05145, hsa05166, hsa05164, hsa05169
PMDEG	Module 1	CDK1	74	Up	Cyclin dependent kinase 1	GO:0010038, GO:1901988, GO:0045930, GO:1901991, GO:0010948, GO:0007093, GO:0007568, GO:0046677, GO:0031145, GO:0000075, GO:0042692, GO:0009123, GO:0009141, GO:0071103, GO:0045927,	hsa04110
PMDEG	Module 1	CDK20	62	Up	Cyclin dependent kinase 20	/	/
PMDEG	Module 1	CCNB1	61	Up	Cyclin B1	GO:0140014, GO:0000819, GO:0000070, GO:0010038, GO:0000280, GO:0007059, GO:0048285, GO:0045839, GO:0098813, GO:0007088, GO:0051784, GO:0051783, GO:0001701, GO:0010965, GO:2000816, GO:0051983, GO:1905819, GO:0051306, GO:0030071, GO:0033048, GO:1905818, GO:0007094, GO:0031577, GO:0071173, GO:0071174, GO:0033044, GO:1902099, GO:0071241, GO:0033046, GO:0007091, GO:0051985, GO:1901988, GO:0045841, GO:0051304, GO:0044784, GO:0071248, GO:1902100, GO:0033047, GO:0033045, GO:0045930, GO:0070482, GO:1901991, GO:0010948, GO:0007093, GO:0036293, GO:0001666, GO:0031145, GO:0051656, GO:0000075, GO:0071453, GO:0042692, GO:0009123, GO:0009141, GO:0048565, GO:0010639, GO:0071103, GO:0045927	hsa04110
PMDEG	Module 2	MTIF2	25	Up	Mitochondrial translational initiation factor 2	GO:0032543, GO:0140053	/
PMDEG	Module 2	MRPS7	25	Down	Mitochondrial ribosomal protein S7	GO:0032543, GO:0140053, GO:0070125, GO:0006414, GO:0022613, GO:0070126	/
PMDEG	Module 3	VEGFA	38	Down	Vascular endothelial growth factor A	GO:0043129, GO:0052547, GO:0048875, GO:0052548, GO:0050900, GO:0010466, GO:0010951, GO:0032103, GO:0010810, GO:0045785, GO:0048732, GO:0001701, GO:0010811, GO:0060249, GO:0033044, GO:0031589, GO:0045807, GO:0097529, GO:0045861, GO:0030595, GO:0070482, GO:0048871, GO:0051346, GO:0036293, GO:0001666, GO:0002576, GO:0001894, GO:0002685, GO:0002688, GO:0060627, GO:0071453, GO:0001954, GO:0042692, GO:0001952, GO:0050920, GO:0030100, GO:0060326, GO:0050678, GO:0002687, GO:0061138, GO:0016049, GO:0045927	hsa05323, hsa04510, hsa04933
PMDEG	Module 3	EGF	34	Up	Epidermal growth factor	GO:0140014, GO:0043129, GO:0052547, GO:0043062, GO:0030198, GO:0000280, GO:0048875, GO:0052548, GO:0048285, GO:0007088, GO:0050900, GO:0010466, GO:0010951, GO:0032103, GO:0010810, GO:0045785, GO:0048732, GO:0051783, GO:0001701, GO:0010811, GO:0060249, GO:0033044, GO:0031589, GO:0045807, GO:0097529, GO:0045861, GO:0030595, GO:0070482, GO:0048871, GO:0051346, GO:0036293, GO:0001666, GO:0002576, GO:0001894, GO:0002685, GO:0002688, GO:0045862, GO:0060627, GO:0071453, GO:0001954, GO:0042692, GO:0007219, GO:0001952, GO:0050920, GO:0030100, GO:0060326, GO:0050678, GO:0051098, GO:0002687, GO:0061138, GO:0016049, GO:0045927	hsa04668, hsa05323, hsa04510, hsa04933

**/:** no significant related GO BP or KEGG pathway term

**Fig. 6:**
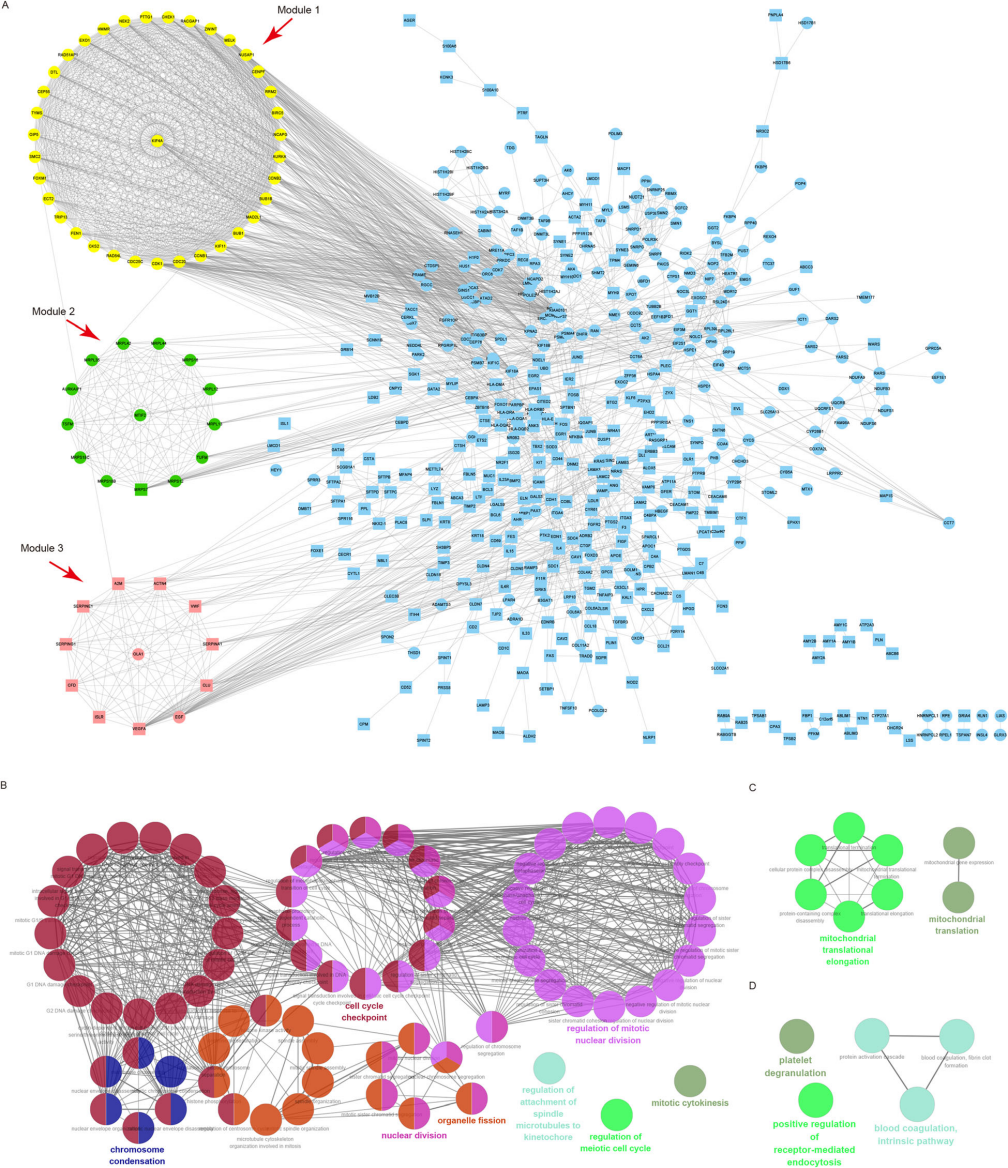
PPI network construction and KEGG pathway analysis of modules. **a** PPI network constructed with the 764 NPDEGs. All cycle nodes stand for upregulated genes, and all square nodes stand for downregulated genes. Genes in module 1 are in yellow, genes in module 2 are in green, genes in module 3 are in pink. Seed nodes live at the center of each module. **b-d** The biological process categories associated with genes in module 1 (**b**), module 2 (**c**), and module 3 (**d**) through GO enrichment analysis (FDR < 0.05). GO, Gene Ontology

There were 521 nodes and 2415 interactions among the 764 PMDEGs, and three significant modules with a score ≥ 10 were screened out via MCODE (Fig. 6a, Supplementary Table S7). Module 1 (score = 32.5) included 36 genes, with CDK1, CDK20 and CCNB1 as hub nodes (Table 3). GO analysis with ClueGO showed that the most significant BP category in this module was enriched in the nuclear division (Fig. 6b, Supplementary Table S8). Module 2 (score = 13.8) included 14 genes, with MTIF2 and MRPS7 as hub nodes (Table 3). The most significant BP category was enriched in the mitochondrial translation (Fig. 6c, Supplementary Table S8). Module 3 (score = 12) included 12 genes, with VEGFA and EG as hub nodes (Table 3). The most significant BP category was enriched in the platelet degranulation (Fig. 6d, Supplementary Table S8).

## Discussion

This study has gained some insights into gene expression modules in osteosarcoma at a genome-wide scale through analyzing three osteosarcoma datasets. A panel of 74 NPDEGs was identified as associated with osteosarcoma tumorigenesis; and 364 PMDEGs were identified as associated with the osteosarcoma metastasis. In addition, it was noticed that seven genes (VAMP8, A2M, HLA-DRA, SPARCL1, HLA-DQA1, APOC1 and AQP1) were continuous upregulating during the oncogenesis and metastasis of osteosarcoma, which suggested that these genes may act as oncogenes and be consistently involved in the pathophysiological process of osteosarcoma. This study further obtained major histocompatibility complex, class II, DR alpha (HLA-DRA) as the hub NPDEGs from the top module with MCODE score = 5, and 7 hub PMDEGs (CDK1, CDK20, CCNB1, MTIF2, MRPS7, VEGFA and EGF) from three top modules with MCODE score > 10. These may be pivotal genes involved in the pathophysiological process of osteosarcoma.

Among these 8 hub genes, HLA-DRA, which is correlated with the procession and presentation of peptide antigen via MHC class II, continued to be up-regulated during the osteosarcoma oncogenesis and metastasis. Apparently, HLA-DRA may have the “driving” function in osteosarcoma. It has been proved as a predictor for metastasis of osteosarcoma [27]. Although until now there is no report about the function and mechanism of HLA-DRA in osteosarcoma, previous studies have shown that HLA-DRA is involved in the evasion of the virus from the immune system [28] and Alzheimer’s disease [29]. Pan Y et al. also listed HLA-DRA as one of the crucial genes in the regulatory network of osteosarcoma they constructed from the dataset GSE28424 [30]. These data indicate that the function of HLA-DRA in osteosarcoma is worthy of attention, especially on the topic of whether it plays a pathophysiological role in osteosarcoma through the process of antigen procession and presentation of peptide antigen via MHC class II. The function enrichment analysis results revealed that HLA class II alleles may be a main impactive factor in osteosarcoma. HLA-DQA1 is also an HLA class II variant that has been reported to be associated with the osteosarcoma risks [31]. Profound understanding of those genes’ immunologic contribution to the etiology of osteosarcoma may be helpful for selecting rational therapeutic targets.

SPARCL1 is an ECM remodeling gene. It modulates extracellular calcium by binding to collagen I [32, 33], which may reveal its potential role in osteosarcoma cell metastasis. Although Zhao SJ et al. [34] reported that SPARCL1 was downregulated in OS by epigenetic methylation of promoter DNA, and that SPARCL1 could suppress osteosarcoma metastasis and recruit macrophages by activation of canonical WNT/β-catenin signaling through stabilization of the WNT-receptor complex, this study, on the contrary, noticed that SPARCL1 continued to be upregulated during osteosarcoma development and metastasis. This contradiction is worthy of further confirmation by collecting clinical samples and expression analysis. Aquaporin 1 (AQP1) is a water-selective transporting protein in cell membranes, and it has been found to be overexpressed in various tumors and promote metastasis and neo-angiogenesis. AQP1 can promote osteosarcoma cell proliferation, adhesion, invasion and tumorigenesis by targeting TGF-β signaling pathway and focal adhesion genes [35], and recruit human BM-MSCs into the osteosarcoma microenvironment [36]. These reports strongly support our current study’s analysis result and confirm that AQP1 is an oncogene and metastasis promoter in osteosarcoma. Although seldom previous studies have revealed the expression and role of VAMP8, APOC1 and A2M in osteosarcoma, exploration in some other tumors has well proved that these genes are important tumor-related regulatory factors [37–39]. However, due to the specificity of osteosarcoma in pathogenesis and signaling pathways involved, additional work is needed to extend the current observation and to clarify the potential causal mechanisms underlying the deregulation of these genes in osteosarcoma.

With regards to these hub genes identified, cyclin-dependent kinase 1 (CDK1) and cyclin-dependent kinase 20 (CDK20) belong to serine/threonine protein kinase family. Cyclin B1 (CCNB1) is a pivotal protein responsible for the control of the cell cycle at the G2/M (mitosis) transition. All the three genes are involved in the cell cycle and growth. Reduction of CDK1 activities is crucial for the survival of osteosarcoma cells [40]. Overexpression of CCNB1 can facilitate the growth rate of osteosarcoma cells and increase their sensitivity to paclitaxel [41]. Several drugs were reported to inhibit cell proliferation or induce cell cycle arrest and apoptosis in human osteosarcoma by downregulating CCNB1 and CDK1 [42–45]. Both mitochondrial translational initiation factor 2 (MTIF2) and mitochondrial ribosomal protein S7 (MRPS7) are proteins implicated in mitochondrial translation. In this study, we have identified MRPS7 and MTIF2 as hub genes involved in the metastasis of osteosarcoma. Mitochondrial translation pathway plays essential roles in programmed cell death. The implication of mitochondria-mediated intrinsic pathway in human osteosarcoma has been observed [46], and inhibition of mitochondrial translation has been reported to be effective and selective in targeting osteosarcoma [47]. Therefore, protein synthesis involved in MRPS7 and MTIF2 within the mitochondrion might also have a potential connection with the development of osteosarcoma. Vascular endothelial growth factor A (VEGFA) is a classic angiogenic factor, which facilitates endothelial proliferation, migration and new vessel formation [48]. Currently, VEGFA has been reported to be very important in evaluating the angiogenesis in osteosarcoma [49]. Inhibition of VEGFA can successfully suppress osteosarcoma growth, metastasis and angiogenesis [50]. All these highlight its therapeutic value in osteosarcoma. Indeed, VEGFA pathway has been prioritized for the development of antiangiogenic therapies in osteosarcoma [51]. Epidermal growth factor (EGF) promotes cell epithelial-mesenchymal transition, metastasis, and progression of osteosarcoma by activating MAPK and PI3K/AKT pathway, which can be blocked by the EGFR-specific inhibitor gefitinib [52]. Thus, EGF-targeting agents should be evaluated to prevent osteosarcoma from deteriorating.

Among the 74 NPDEGs identified, notable dysregulation of gene expression was observed clustered in immune related diseases, phagocytosis, antigen procession and presentation. Bone resorption is accomplished by osteoclasts, which can be seen as highly specialized macrophages [53]. Thus, bone microenvironment represents a unique compartment of the immune system, in which immunological cytokines form part of an intercellular crosstalk that is relevant to the development of osteosarcoma [54, 55]. Osteosarcoma cells control the recruitment and differentiation of immune infiltrating cells and establish a local immune tolerant environment that is favorable to the tumor growth [56]. This is in agreement with the current demonstration that those NPDEGs in osteosarcoma are clustered in multiple immune diseases and T helper cells differentiation. Besides, osteoblasts can express major histocompatibility complex II (MHC class II) to present antigen [4]. Thus, deregulation of genes involved in antigen presentation may be an early event in osteosarcoma oncogenesis. MHC II is only expressed on the surface of antigen presenting cells (APC), such as macrophages, dendritic cells and B cells. APC presents exogenous peptides or endogenous peptides to helper T cells by binding MHC-II to peptides, and thus informs that the body is being invaded [57]. Previous studies have shown that osteosarcoma cells can express moderate to high levels of Herpes virus entry mediator on the tumor [58], and osteosarcoma cells can be killed and phagocytosed by Killer Dendritic Cells [59]. Therefore, during the process of malignant transformation, osteosarcoma cells express some antigenic substances, which are recognized by APC and presented to helper T cells via MHC-II. In this way, APC helps to connect innate and adaptive immunity to tumor. These suggest that MHC-II mediates immune responses in the tumor microenvironment, thus it could be an alternative target for novel immune therapies and targeting antigen presentation may be clinically valuable in early intervention.

Among the 764 PMDEGs, notable dysregulation of gene expression was observed in well-known metastatic related pathways including CAMs, Focal adhesion and ECM-receptor interaction. It was also found that the tumor necrosis factor (TNF) signaling pathway, which is always activated in human osteosarcoma cells [60], was significantly correlated to osteosarcoma metastasis. Hence, to abnormalize the function of the TNF signaling pathway might be a potential target for chemotherapy of advanced osteosarcoma [61]. Interestingly, cell cycle is also the key signal involved in osteosarcoma metastasis. Previous reports have revealed that cell cycle and apoptosis are two major dysregulated events in human malignancy cells [62]. Evolution of cancer is a complex process. Potentially oncogenic proliferative signals can couple to the induction of apoptosis, which restricts subsequent clonal expansion and neoplastic evolution. However, tumor progression occurs when these growth-inhibitory mechanisms are thwarted by compensatory mutations. Deregulated cell proliferation and the obligate compensatory suppression of apoptosis provide a minimal ‘platform’ that is necessary to support further neoplastic progression, which in turn propels the tumor cell and its progeny into uncontrolled expansion and invasion [62].

The limitations of this study also should be recognized. First of all, when analyzing the DEGs, in view of the complexity of datasets in the study, it is impossible to consider all important factors—for example, different ages, races, regions, cell lineage as well as tumor stages and classification of patient. Secondly, according to the results, all the seven genes, which are continuously deregulating during the oncogenesis and metastasis of osteosarcoma, are actually up-regulated ones. Yet, the mechanism of upregulation has not been clear. Therefore, more evidences are required to find out the biological foundation. Finally, this study mainly focuses on analyzing the expression levels of genes involved in tumorigenesis and metastasis. Some of these genes have been reported as biomarkers for osteosarcoma, while the role of HLA-DRA, MTIF2, MRPS7 and CDK20, should also be further systematically investigated based on actual diseased tissues or even cell lines and animal models.

In conclusion, this study identified several DEGs that may be involved in the carcinogenesis and metastasis of osteosarcoma through comprehensive bioinformatics analyses, and unveiled a series of hub genes and pathways. However, further experimental studies are needed to elucidate the biological function and underlying mechanism of these genes in osteosarcoma.

## Supplementary information

**Additional file 1 Supplementary Table S1.** Dysregulation genes between osteosarcoma and normal samples.

**Additional file 2 Supplemental Table S2**. Functional Enrichment Analysis of DEGs between osteosarcoma and normal samples.

**Additional file 3 Supplementary Table S3.** Dysregulation genes between no-metastatic and metastatic osteosarcoma samples.

**Additional file 4 Supplemental Table S4**. Functional Enrichment Analysis of DEGs between primary and metastatic osteosarcoma samples.

**Additional file 5 Supplementary Table S5**. Characteristics of all nodes in the PPI network of the NPDEGs.

**Additional file 6 Supplementary Table S6**. Biological pathway enrichment analysis of the NPDEGs in the module 1.

**Additional file 7 Supplementary Table S7**. Characteristics of all nodes in the PPI network of the PMDEGs.

**Additional file 8 Supplementary Table S8**. Biological pathway enrichment analysis of the PMDEGs in these modules.

**Additional file 9 Supplementary Table S9**.

## Data Availability

The datasets generated and analyzed during the current study are available in the GEO repository (https://www.ncbi.nlm.nih.gov/geo/).

## Abbreviations

CAMs: Cell adhesion molecules
DEGs: Differentially expressed genes
GEO: Gene Expression Omnibus
GO: Gene Ontology
KEGG: Kyoto Encyclopedia of Genes and Genomes
MCODE: Molecular Complex Detection
STRING: Search Tool for the Retrieval of Interacting Genes
A2M: Alpha-2-Macroglobulin
APOC1: Apolipoprotein C1
AQP1: Aquaporin 1
CCNB1: Cyclin B1
CDK1: Cyclin-dependent kinase 1
CDK20: Cyclin-dependent kinase 20
ECM: Extracellular matrix
EGF: Epidermal growth factor
HLA-DQA1: Major Histocompatibility Complex, Class II, DQ Alpha 1
HLA-DRA: Major histocompatibility complex, class II, DR alpha
MTIF2: Mitochondrial translational initiation factor 2
MRPS7: Mitochondrial ribosomal protein S7
SPARCL1: SPARC Like 1
TNF: Tumor necrosis factor
VEGFA: Vascular endothelial growth factor A
VAMP8: Vesicle Associated Membrane Protein 8
