# Supplementary material for: Identification of potential crucial genes and key pathways in osteosarcoma
Source: Hereditas. 2020 Jul 14;157:29. doi: 10.1186/s41065-020-00142-0 (PMC7362476; doi:10.1186/s41065-020-00142-0)
Supplement: Supplementary file 1 — Additional file 1 Supplementary Table S1. Dysregulation genes between osteosarcoma and normal samples. [file 41065_2020_142_MOESM1_ESM.docx]

**Supplementary Table 1 Dysregulation genes between osteosarcoma and normal samples**

| **Regulation** | **DEGs (gene symbol)** |
| --- | --- |
| Up-regulated | VAMP8, HLA-DRB4, SEMA4D, FXYD6, RNASE6, A2M, SLCO2B1, HLA-DRA, EDNRA, LY86, GMFG, FGFR3, CD53, CD300A, TCF4, MS4A6A, LEF1, SLA, HLA-DPA1, CPVL, EFNA1, COL5A2, HLA-DMB, SPP1, MXRA5, FOLR2, GIMAP6, AIF1, CD86, HLA-DQA1, COLEC12, RGS1, CD14, ARHGDIB, CD163, FCGR2A, TYROBP, PECAM1, ACP5, GIMAP4, IFI30, CD74, SPARCL1, ELMO1, CA2, CYBB, C1QB, HBB, HEY1, AQP1, RNASE1, LAPTM5, LCP1, CPE, FCER1G, C1QA, HLA-DRB3, APOC1, HCLS1, MEF2C, HBA1 |
| Down-regulated | GCLM, ARID5B, PERP, FST, DUSP14, FHL2, CRIM1, KCTD9, NQO1, TXNRD1, SERPINE1, DRAP1, FOSL1 |
