# Supplementary material for: Identification of potential crucial genes and key pathways in osteosarcoma
Source: Hereditas. 2020 Jul 14;157:29. doi: 10.1186/s41065-020-00142-0 (PMC7362476; doi:10.1186/s41065-020-00142-0)
Supplement: Supplementary file 3 — Additional file 3 Supplementary Table S3. Dysregulation genes between no-metastatic and metastatic osteosarcoma samples. [file 41065_2020_142_MOESM3_ESM.docx]

**Supplementary Table 3 Dysregulation genes between no-metastatic and metastatic osteosarcoma samples**

| **Regulation** | **DEGs (gene symbol)** |
| --- | --- |
| Up-regulated | CYCS, IGF2BP3, CCNB1, NDUFS1, TTC37, GINS1, STK26, TIGAR, KPNA2, NUDT1, BIRC5, FAM98A, FOXM1, HSPD1, CETN3, CDK1, NUP37, EIF3M, MRPS12, HIST1H2BJ, WDR12, NOC3L, MAD2L1, GJA9, KIF4A, EIF2S1, SNORD45B, PRKDC, TYMS, MELK, RABGGTB, SNRPG, XPOT, ZWINT, POP4, CKS2, NDUFB3, GGH, LMNB1, PCLAF, PSMB7, MRPL13, CCNB2, RMDN1, CEP55, CTPS1, HSPE1, CDK7, HEY1, RAD51AP1, DDX1, FRG1JP, SNORD45C, CDC23, MRPL42, MTIF2, PHB, ZC3H15, PCOLCE2, NME1, MRPS18B, CCT6A, NUDT21, NEK2, RNASEH1, CNPY2, MMP13, GOLM1, RFC3, YARS2, RSL24D1, KIF11, KRAS, EMG1, HSPA4, NRAS, CERKL, SNRPD1, SMN2, LRPPRC, MRPL35, CDC20, SRP19, BUB1, TRIP13, RAN, PSMD14, ITGB3BP, ITGA4, PSMA4, MCTS1, CHCHD3, SMC2, NAP1L3, TFB2M, ATAD2, LSM5, TDG, PUS7, EEF1E1, CEP76, RRM2, FEN1, HPRT1, NCAPD2, FRG1CP, STOML2, BUB1B, SLC30A5, SMN1, DTL, WDR41, PAICS, HMMR, MCM6, CCT5, RARS, SNORD45A, NMD3, OLA1, RPL26L1, NCAPG, NDC1, CENPF, NUSAP1 |
| Down-regulated | BTG2, VCY1B, TCL6, CEACAM6, ITGA3, GGT2, IGH, GRK5, SEL1L3, MAOA, APOE, ALDH2, SLCO2A1, LAMP3, SLC8B1, CD82, SERPING1, LOC102724788, CLEC3B, RHOBTB2, PNPLA6, GFI1, RBPMS, ACSF2, PPL, CSRP1, ZFP36, NDRG2, IL23A, CYP2B7P, CCL14, ANXA8, KRT8, KANK3, GGTLC2, EHD2, CD2, IGHA2, HMBOX1, RAMP3, LAMA3, TNFAIP3, FOXO1, TNFSF13, ATP2A3, SFTPA1, FMNL1, C4B, GGT1, LRRC32, CCL18, SCGB1A1, CA4, ISLR, PTPRF, CXCL2, NR4A1, AQP3, TPSB2, TACSTD2, CPA3, CBX7, VCY, CABIN1, FBP1, KRT18, SFTPC, EPS8L2, GGTLC1, IL32, LSR, LAMC2, GATA2, MARCO, C4BPA, CEACAM1, EPAS1, CYB5A, HSD17B6, ITIH4, APOC1, ABLIM1, TRADD, CD1C, VAMP2, HCP5, PTK2B, TGM2, CRYM, TIMP3, MIR8085, MINK1, SOD3, MUC1, TBX2, REC8, C4A, SERPINA1, CLDN7, CPB2, CTF1, EGFL7, SLC34A1, LTBP2, C4B_2, HPR, TSPYL2, ZYX, LMOD1, ZBTB16, S100A14, ABCC3, MYH11, DUSP1, NPR3, SLPI, GPRC5A, TNS1, ANG, G0S2, UBD, C7, TXNIP, CCL15-CCL14, TNFSF12-TNFSF13, TPSAB1, CLDN5, MAOB, PTGDS, MYRF, ANXA8L1, DMBT1, SDC1, CDH1, CYP27A1, SPOCK2, CLU, PRODH, EPHX1, CD52, ICAM1, SPINT1, IGHA1, LTF, SFTPB, METTL7A, CEBPA, TSPAN1, CCL21, FMO2, SFTPA2, PLLP, COBL, PGC, FCN3, CYP2B6, GPX3, ANK3, BCL3, ABLIM3, PRSS8, CTSH, PLAC8, MLPH, SPINT2, CES1, MISP, FBLN5 |
